# Clinical Efficacy of a Single Two Gram Dose of Azithromycin Extended Release for Male Patients with Urethritis

**DOI:** 10.3390/antibiotics3020109

**Published:** 2014-04-02

**Authors:** Satoshi Takahashi, Hiroshi Kiyota, Shin Ito, Akihiko Iwasawa, Yoshiki Hiyama, Teruhisa Uehara, Koji Ichihara, Jiro Hashimoto, Naoya Masumori, Kenichi Sunaoshi, Koichi Takeda, Nobukazu Suzuki, Takahide Hosobe, Hirokazu Goto, Hidenori Suzuki, Shoichi Onodera

**Affiliations:** 1 Department of Urology, Sapporo Medical University School of Medicine, S. 1, W. 16, Chuo-ku, Sapporo, Hokkaido 0608543, Japan; E-Mails: mayahi55@hotmail.com (Y.H.); teruhisa5963@yahoo.co.jp (T.U.); kichi@sapmed.ac.jp (K.I.); jiroh@sapmed.ac.jp (J.H.); masumori@sapmed.ac.jp (N.M.); 2 Department of Urology, the Jikei University Katsushika Medical Center, 6-41-2 Aoto Katsushika-ku, Tokyo 1258506, Japan; E-Mail: kiyota@jikei.ac.jp; 3 iClinic, 5-9-6, Nagamachi, Taihaku-ku, Sendai, Miyagi 9820011, Japan; E-Mail: itsn.icl@gmail.com; 4 Iwasawa Clinic, Sapporo Medical University School of Medicine, S. 1, W. 16, Chuo-ku, Sapporo, Hokkaido 0600061, Japan; E-Mail: iwasawa@mb.snowman.ne.jp; 5 Teine Urologic Clinic, 1-12, Maeda 6-7, Teine-ku, Sapporo 0060816, Japan; E-Mails: k1sunaoshi@yahoo.co.jp (K.S.); k-takeda@teine-uro.or.jp (K.T.); n-suzuki@teine-uro.or.jp (N.S.); 6 Hosobe Clinic, 1-1-15, Nezu, Bunkyo-ku, Tokyo 1130031, Japan; E-Mail: urohoso428@gmail.com; 7 Department of Urology, Fuji City General Hospital, 50 Takashima-Cho, Fuji, Shizuoka 4178567, Japan; E-Mails: gotou_hirokazu@ss.city.fuji.shizuoka.jp (H.G.); hidenori-s@sf.tokai.or.jp (H.S.); onodera_shouichi@sa.city.fuji.shizuoka.jp (S.O.)

**Keywords:** azithromycin, extended release, urethritis, *Neisseria gonorrhoeae*, *Chlamydia trachomatis*, *Mycoplasma genitalium*, *Ureaplasma urealyticum*

## Abstract

To clarify the clinical efficacy of a single oral 2 g dose of azithromycin extended-release for heterosexual male patients with urethritis, and the current antimicrobial sensitivity of *Neisseria gonorrhoeae* to azithromycin, a prospective clinical trial was conducted from 2011–2013. In patients with gonococcal urethritis, the eradication rate was 90.9% (30 of 33). The susceptibility rates of isolated *Neisseria gonorrhoeae* strains to ceftriaxone, spectinomycin, cefixime and azithromycin were 100%, 100%, 95.3% (41/43) and 37.2% (16/43), respectively. In the patients with nongonococcal urethritis, the eradication rate was 90.0% (45 of 50). The microbiological eradication rates for the pathogens were 90.9% (30/33) for *Neisseria gonorrhoeae*, 91.5% (43/47) for *Chlamydia trachomatis*, 71.4% (5/7) for *Mycoplasma genitalium*, and 100% (13/13) for *Ureaplasma urealyticum*. The main adverse event was diarrhea and its manifestation rate was 35.2% (32 of 120). The symptom of diarrhea was mostly temporary and resolved spontaneously. The conclusion was that the treatment regimen with a single oral 2 g dose of azithromycin extended-release would be effective for patients with urethritis. However, the antimicrobial susceptibilities of *Neisseria gonorrhoeae* and *Mycoplasma genitalium* should be carefully monitored because of possible treatment failure.

## 1. Introduction

The pathogen of gonococcal urethritis (GU) is *Neisseria gonorrhoeae* and the principal p athogens of nongonococcal urethritis (NGU) are *Chlamydia trachomatis*, *Mycoplasma genitalium*, *Ureaplasma urealyticum* [1]. Clinical guidelines clearly indicate different treatment regimens for male patients with GU and NGU. The guideline published by the Centers for Disease Control and Prevention (CDC), USA, recommends a treatment regimen with both a single 250 mg dose of ceftriaxone intramuscularly and a single 1 g dose of azithromycin (AZM) orally for patients with GU [2], and a regimen with a single 1 g dose of AZM orally for patients with NGU [3]. Therefore, in the current clinical situation, AZM is a key drug worthy of note. However, several recent reports showed that strains of *N. gonorrhoeae* [4,5,6] and *M. genitalium* [7,8] highly resistant to AZM occurred worldwide. In addition, recent clinical trials revealed the decreased efficacy of a 1 g dose of AZM against NGU [9].

AZM is a macrolide antimicrobial agent and its ideal treatment regimen should achieve a higher area under the curve (AUC) above minimal inhibitory concentrations (MICs) value due to the current theory on pharmacokinetics (PK) and pharmacodynamics (PD) [10]. However, the conventional type of AZM, called immediate release (IR), has an increased risk of adverse events if patients are treated with a dose of AZM IR 2 g [11]. To overcome this situation, a single 2 g dose of AZM extended release (ER) has been developed [12]. AZM ER microsphere formulation can delay the release of AZM and bypass the upper gastrointestinal motilin receptors. In addition, this formulation can decrease the release rate of AZM from the matrix which leads to reduced concentration-dependent local irritation on upper gastrointestinal mucosa. In Japan, AZM ER is available to treat patients with both GU and NGU because it is officially approved, although AZM ER is not available for patients with urethritis in most countries outside of Japan. Therefore, there have been few studies about the clinical efficacy of AZM ER for male patients with urethritis. The aim of this study was to clarify the clinical efficacy of single 2 g dose of AZM ER for male patients with GU and NGU, as well as the current antimicrobial efficacy of AZM against *N. gonorrhoeae*.

## 2. Patients and Methods

### 2.1. Study Design

This prospective, multi-institutional, open label, single-arm clinical study was conducted from September 2011 to August 2013. It was designed essentially according to Japanese guidelines [13].

### 2.2. Patients

This clinical study included heterosexual male patients with both GU and NGU who were 20 years old or older. Diagnosis of GU was done based on both symptoms, urethral pain and pus discharge on external urethral meatus, suspecting urethritis, suspecting urethritis [14] and positive *N. gonorrhoeae* detected by microscopic examination of Gram staining, culture of urethral pus discharge or a nucleic acid amplification test (NAAT) of first-voided urine (FVU). Diagnosis of NGU was done based on both symptoms suspected to be urethritis and negative *N. gonorrhoeae* examined by microscopic examination of culture of urethral pus discharge or commercially available NAAT of FVU. In addition, *C. trachomatis*, *M. genitalium* and *U. urealyticum* were also detected by a microbiological test using NAAT. The patients diagnosed with GU or NGU were treated with a single 2 g dose of AZM ER. 

### 2.3. Procedures for Detection of Pathogens

At each clinic, pus discharge from the external urethral meatus was collected as the specimen for Gram staining and/or culture of *N. gonorrhoeae*. The culture method was described in detail in a previous report [15]. In brief, the specimen was applied on Thayer-Martin Selective Agar (Becton Dickinson, Cockeysville, MD, USA) and immediately transported in a Bio-Bag Environmental Chamber Type C (Becton, Dickinson) to Mitsubishi Chemical Medience Corporation, Tokyo. Then, it was incubated at 35 °C for 48 h in a 5% CO_2_ atmosphere.

About 20 mL of FVU was taken from each patient and used as a specimen. *C. trachomatis* and *N. gonorrhoeae* were detected with the commercially available NAAT test kit by transcription-mediated amplification, polymerase chain reaction (PCR) or strand displacement amplification. *M. genitalium* and *U. urealyticum* were detected using a previously reported PCR method at Mitsubishi Chemical Medience Corporation [16]. 

### 2.4. Antimicrobial Susceptibility Testing

In this study, the minimum inhibitory concentrations (MICs) of eight antimicrobial agents were determined: penicillin G (PCG) (Wako Pure Chemical Industries, Osaka, Japan), ceftriaxone (CTRX) (Wako Pure Chemical Industries), cefodizime (CDZM) (Taiho Pharmaceutical Co., Tokyo, Japan), cefixime (CFIX) (Wako Pure Chemical Industries), spectinomycin (SPCM) (Wako Pure Chemical Industries), azithromycin (AZM) (LKT Laboratories, St. Paul, MN, USA), ciprofloxacin (CPFX) (Tokyo Chemical Industry Co., Tokyo, Japan) and levofloxacin (LVFX) (Tokyo Chemical Industry Co.). The MICs were determined by an agar dilution method according to a report (M07-A9 and M100-S23 Package) from the Clinical and Laboratory Standards Institute (CLSI). After incubation at 35 °C for 20 h in a 5% CO_2_ atmosphere, the lowest concentration of the antimicrobial agent that could inhibit bacterial growth completely was defined as the MIC. In addition, the lowest concentration of the drug that could inhibit approximately 90% and 50% of strains was defined as the MIC_90_ and MIC_50_, respectively. According to the criteria of the CLSI (M100-S23), the MIC breakpoints for susceptibility and resistance were ≦0.06 μg/mL and ≧2 μg/mL for penicillin, respectively. For CTRX and CFIX the breakpoint for susceptibility was ≦0.25 μg/mL. For SPCM and CPFX the susceptibility and resistance breakpoints were ≦32 μg/mL and ≧128 μg/mL, and ≦0.06 μg/mL and ≧1 μg/mL, respectively. The MIC breakpoint of AZM was not defined; however, for it we used the MIC breakpoint for susceptibility and resistance of ≦0.25 μg/mL and ≧1 μg/mL, respectively, according to the European Committee on Antimicrobial Susceptibility Testing (EUCAST [17]). The MIC breakpoints of CDZM and LVFX were not defined either; therefore, we used the MIC breakpoint for susceptibility and resistance of CDZM of ≦0.06 μg/mL and ≧0.5 μg/mL, and those of LVFX were ≦0.12 μg/mL and ≧2 μg/mL, respectively.

### 2.5. Outcome

We evaluated both microbiological and clinical cure outcomes judged 1–4 weeks after the completion of AZM ER treatment. The definition of each microbiological and clinical cure was essentially using the Japanese guideline for clinical research. In the Japanese guideline [13], the optimal time to evaluate bacteriological and clinical cure outcomes is 2–4 weeks post-treatment; however, we modified the duration for judgment because AZM ER was given as a single dose.

In the patients with GU, the microbiological outcome was the primary endpoint or assessment, and eradication meant no *N. gonorrhoeae* detected by culture or by NAAT posttreatment. In those with GU, failure of microbiological outcome meant *N. gonorrhoeae* detected by culture or modification of treatment, including a change or addition of antimicrobial agents. In those patients, clinical cure meant no symptoms derived from GU.

In the patients with NGU, the microbiological outcome was the primary endpoint or assessment, and eradication meant no *C. trachomatis*, *M. genitalium*, or *U. urealyticum* detected by NAAT posttreatment. In those with NGU, failure as microbiological outcome meant *C. trachomatis*, *M. genitalium* or *U. urealyticum* detected by culture or modification of treatment including a change or addition of antimicrobial agents. In those patients, clinical cure meant no symptoms derived from NGU and failure meant continuation of a symptom derived from urethritis or with changes/additions of antimicrobial agents post-treatment. Only the clinical outcome was analyzed in the patients without any microbial detection. 

### 2.6. Assessment of Adverse Events

At the second visit, the doctors interviewed each patient in detail about whether adverse events, including diarrhea, abdominal pain, nausea and so on, had occurred. The grade of adverse events was determined according to Common Terminology Criteria for Adverse Events (CTCAE) v4.0. 

### 2.7. Ethical Considerations

This clinical study design was approved by Institutional Review Board of Sapporo Medical University Hospital ([18]; Nos. 22–128, 24–3011) and written informed consent was obtained from each subject. This clinical study is registered in the University Hospital Medical Information Network Clinical Trial Registry (UMIN-CTR [19]; UMIN ID; UMIN000006359).

## 3. Results

**Figure 1:**
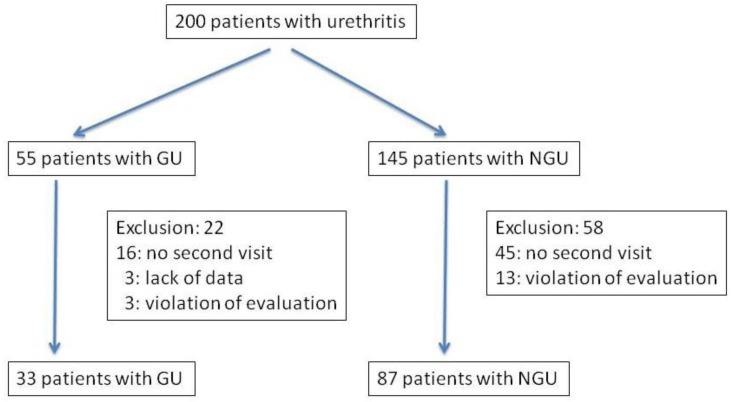
Flow chart for assessment of patients: gonococcal urethritis = GU, nongonococcal urethritis = NGU).

A total of 200 patients were included in this study (Figure 1). Of these 200 patients, 55 were diagnosed with GU, including concomitant, 10 positive for *C. trachomatis*, one with *M. genitalium* and two with *U. urealyticum*. There were 43 *N. gonorrhoeae* strains isolated from the patients with GU and the MICs against these strains were measured. The microbiological and clinical efficacy were analyzed in 33 patients with GU including for analysis and eight patients of concomitant urethritis with *C. trachomatis*, one of urethritis with *M. genitalium* and one with *U. urealyticum*. In these 33 patients with GU, median age was 33 (range: 21–50) years old. The median duration from the completion of treatment to the second visit was 9 (range: 7–27) days. Twenty-two patients with GU were excluded from this study, because of no second visit for 16, lack of data for three and violation of the evaluation period in three. There were 145 patients who were diagnosed as having NGU. Of these 145 patients, 58 were excluded from this study, because of no second visit for 45 and violation of the evaluation period for 13. Finally, 87 could be analyzed, and they were classified into 35 cases of urethritis with *C. trachomatis*, three with *M. genitalium*, five with *U. urealyticum*, four with *C. trachomatis* and *U. urealyticum*, three with *M. genitalium* and *U. urealyticum*, and 37 without any microbial detection. In these 87 patients with NGU, median age was 33 (range: 20–65) years old. The median duration from the completion of treatment to the second visit was 9 (range: 7–27) days. Of those patients included in the analysis, none had sexual intercourse between the first and second visits and, based on the interviews, they took the drug as prescribed.

The MIC of each antimicrobial agent was measured for 43 *N. gonorrhoeae* strains (Table 1). The rate of susceptibility to PCG was 9.3% (4/43) and that for CPFX or LVFX was 20.9% (9/43). The rates for CTRX, SPCM and CFIX were 100%, 100% and 95.3% (41/43), respectively. That for AZM was 37.2% (16/43).

In the patients with GU, the eradication rate, as the microbiological outcome, was 90.9% (30 of 33) and the cure rate, as the clinical outcome, was 84.8% (28 of 33). Of the three patients with GU that could not be eradicated by a single 2 g dose of AZM ER, one was treated by a single 1 g dose of intravenous CTRX and his pathogen was eradicated. The other two patients could not be followed-up further. The symptom of pus discharge had not disappeared in any of the three patients at the second visit. The MICs of AZM ER in these three patients with microbiological failure were 0.5 μg/mL, 1 μg/mL, and 2 μg/mL (Table 2). If an MIC with complete eradication is defined as the MIC breakpoint of susceptibility, 0.25 μg/mL was the MIC breakpoint for AZM in this study. In one patient with GU, *N. gonorrhoeae* was eradicated; however, *M. genitalium* was not eradicated. In the patients with NGU, the microbiological eradication rate was 90.0% (45 of 50) and the clinical cure rate was 94.3% (82 of 87) (Table 3). Four patients with treatment failure of *C. trachomatis*-positive urethritis did not complain of symptoms related to urethritis at the second visit. Three patients did not visit the clinic again and no further follow-up could be done. Only one patient visited again 60 days after the first visit and was treated with minocycline successfully.

**Table 1:** The MIC distribution of each antimicrobial agent against 43 clinically isolated *N. gonorrhoeae* strains.

Antimicrobial agents	MICs (µg/mL)																
	≦0.001	0.002	0.004	0.008	0.015	0.03	0.06	0.12	0.25	0.5	1	2	4	8	16	32	64
PCG	-	-	-	-	2	-	2	9	7	9	6	6	1	-	-	1	-
CTRX	2	4	6	6	5	11	7	2	-	-	-	-	-	-	-	-	-
CDZM	-	1	3	8	2	9	11	8	1	-	-	-	-	-	-	-	-
CFIX	-	1	1	6	5	2	9	14	3	2	-	-	-	-	-	-	-
SPCM	-	-	-	-	-	-	-	-	-	-	-	-	-	8	22	13	-
AZM	-	-	-	-	1	-	6	5	4	9	11	5	1	-	1	-	-
CPFX	-	-	3	4	2	-	-	-	-	-	-	1	-	4	8	17	4
LVFX	-	-	3	4	2	-	-	-	-	-	1	1	4	18	9	1	-

PCG: penicillin G; CTRX: ceftriaxone; CDZM: cefodizime; CFIX: cefixime; SPCM: spectinomycin; AZM: azithromycin; CPFX: ciprofloxacin; LVFX: levofloxacin.

The microbiological eradication rates of the pathogens were 90.9% (30/33) for *N. gonorrhoeae*, 91.5% (43/47) for *C. trachomatis*, 71.4% (5/7) for *M. genitalium*, and 100% (13/13) for *U. urealyticum*. 

The interview at the second visit showed that diarrhea was the main adverse event and its manifestation rate was 35.2% (32 of 120). However, this diarrhea was mostly temporary and no further additional treatment was necessary for almost all the patients with diarrhea. In the patients having temporary diarrhea, the symptom was resolved within one day when the patient took AZM ER. Therefore, the degree of this adverse event was less than grade 1 defined by the CTCAE.

**Table 2:** The MIC distribution of each antimicrobial agent against 26 clinically isolated *N. gonorrhoeae* strains in the patients with GU for whom it was possible to judge microbiological outcomes. Eradication means no *N. gonorrhoeae* detected by culture after treatment with AZM ER. Failure means *N. gonorrhoeae* detected by culture after treatment with AZM ER.

Antimicrobial agents	Outcome	MICs																
		≦0.001	0.002	0.004	0.008	0.015	0.03	0.06	0.12	0.25	0.5	1	2	4	8	16	32	64
PCG	Eradication	-	-	-	-	2	-	1	5	5	4	4	2	-	-	-	-	-
	Failure												1	1			1	
CTRX	Eradication	1	3	2	3	4	2	6	2	-	-	-	-	-	-	-	-	-
	Failure				1		1	1										
CDZM	Eradication	-	1	2	4	-	3	7	5	1	-	-	-	-	-	-	-	-
	Failure						1		2									
CFIX	Eradication	-	1	1	3	2	-	3	8	3	2	-	-	-	-	-	-	-
	Failure						1	1	1									
SPCM	Eradication	-	-	-	-	-	-	-	-	-	-	-	-	-	3	14	6	-
	Failure														1		2	
AZM	Eradication	-	-	-	-	-	-	4	3	3	6	6	1	-	-	-	-	-
	Failure										1	1	1					
CPFX	Eradication	-	-	1	2	2	-	-	-	-	-	-	1	-	4	4	8	1
	Failure																3	
LVFX	Eradication	-	-	1	2	2	-	-	-	-	-	1	1	4	8	4	-	-
	Failure															3		

**Table 3:** Microbiological and clinical outcomes in patients with gonococcal urethritis (GU) and nongonococcal urethritis (NGU).

**Urethritis**	**Pathogen coinfection**	**Number**	**Eradication** **(Number)**	**Cure (Number)**	**Pathogen of treatment failure and number**
GU	None	23	30	28	*M. genitalium* in 1
	*C. trachomatis*	8			
	*M. genitalium*	1			
	*U. urealyticum*	1			
**Urethritis**	**Pathogen infection**	**Number**	**Eradication**	**Cure**	**Pathogen in failure and number**
NGU	*C. trachomatis*	35	31	34	*C. trachomatis* in 4
	*M. genitalium*	3	2	3	
	*U. urealyticum*	5	5	5	
	*C. trachomatis* and *U. urealyticum*	4	4	4	
	*M. genitalium* and *U. urealyticum*	3	3	3	
	Without any microbial detection	37	-	33	

## 4. Discussion

There have been several critical issues concerning how to treat patients with GU or NGU properly. Among these, the occurrence and development of drug-resistant *N. gonorrhoeae* strains has been the focus of worldwide attention [20]. The updated treatment regimen recommended by the CDC of the USA for patients with GU is CTRX 250 mg in a single muscular dose plus AZM 1 g orally in a single dose or doxycycline 100 mg orally twice daily for seven days [2]. In Japan, the guidelines of the Japanese Society for Sexually Transmitted Infections (STI), the Japanese Association for Infectious Diseases and Japanese Society of Chemotherapy commonly recommend a treatment regimen using CTRX 1 g in a single intravenous administration, CDZM 1 g in a single intravenous administration or SPCM 2 g in a single intramuscular dose. CTRX has been a key drug for the treatment of GU; however, the Gonococcal Isolate Surveillance Project (GISP) (2000–2010) in the USA showed that the frequencies of *N. gonorrhoeae* isolates with CFIX MICs ≧0.25 μg/mL and CTRX MICs ≧0.125 μg/mL have been increasing gradually [21]. Although the prevalence of *N. gonorrhoeae* isolates with elevated MICs remains low, the susceptibility rates to CFIX and CTRX are decreasing. In addition, a CTRX-resistant *N. gonorrhoeae* strain was isolated in 2009 in Kyoto, Japan [22]. Thus, in the current situation there will inevitably be a need for alternative effective treatment regimens for GU.

AZM is a macrolide antimicrobial agent that has efficacy against pathogens of urethritis [23]. In general, 1 g of AZM immediate release (IR) is widely used for the treatment of urethritis according to several guidelines [3]. Although the treatment efficacy with AZM IR 1 g orally is good [23], according to the principles of PK/PD, a greater AUC/MIC could probably obtain better treatment efficacy by a macrolide [10]. However, a relatively high frequency of gastrointestinal adverse events could occur in patients treated with AZM IR 2 g orally in a single dose [11], although the treatment efficacy of AZM IR 2 g is as good as that of CTRX 250 mg intramuscular administration in patients with GU [11]. Therefore, efforts have been made to achieve a greater AUC/MIC with AZM ER and to reduce adverse events [12]. According to a report from India, the AZM IR 2 g single dose regimen achieved 100% microbiological efficacy for patients with GU [24]. 

In this study, the microbiological eradication rate was 90.9% in the patients with GU. This eradication rate was relatively low compared to those in previous reports [21]. The MIC distribution of AZM also shifted to higher MICs and the rate of susceptibility to AZM was 37.2% if the MIC breakpoint of susceptibility was defined as 0.25 μg/mL. This report from Japan suggests that the susceptibility to AZM might be decreasing, though two recent reports showed that the susceptibility rate was high, with no resistant strains identified [25,26]. In our study, three patients with GU had treatment failure, with *N. gonorrhoeae* strains having MICs of 0.5 μg/mL, 1 ug/mL and 2 μg/mL. In these MIC groups, six patients with an MIC of 0.5 μg/mL, six with 1 ug/mL and one with 2 μg/mL were treated successfully and the *N. gonorrhoeae* strains were completely eradicated. This study suggested that the AZM ER 2 g single dose treatment regimen is promising and adequate to eradicate *N. gonorrhoeae*. This result suggested that future guidelines should limit the treatment to AZM IR 1 g. However, recent reports from the USA and Europe revealed that the susceptibility of *N. gonorrhoeae* to AZM has been decreasing and highly resistant isolates were identified in the USA [4,5,6,27]. Therefore, a nationwide surveillance system such as the GISP or the surveillance system of the Japanese Association for Infectious Diseases, Japanese Society of Chemotherapy and the Japanese Society for Clinical Microbiology, is indispensable for the management of GU. In addition, it is important to check the MIC breakpoint of susceptibility because a higher AUC/MIC due to the AZM ER 2 g single dose may contribute to a better outcome than treatment with AZM IR 1 g.

The treatment regimen using a single 1 g oral dose of AZM IR is a suitable recommendation for patients with NGU [21]. In this study, the treatment efficacy of the single 2 g AZM ER oral dose was excellent and the results were similar to those of our previous reports [23,28,29,30]. Therefore, this treatment regimen can also be strongly recommended for patients with NGU because of its high treatment efficacy as well as being an ideal dose based on PK/PD principles. However, there has been an issue about treatment failure against *M. genitalium* recently [8,9]. In our study, the microbiological eradication rate was 71.5% (5/7) for *M. genitalium*, although the sample number was small. A recent report from the USA showed that treatment failure was common among patients with *M. genitalium*-positive NGU who received a single 1 g dose of AZM IR [9]. In addition, highly AZM-resistant *M. genitalium* has been isolated from STI patients [27]. Because the sample number was limited in this study, the definitive treatment efficacy of AZM ER 2 g for patients with *M. genitalium*-positive NGU remains to be determined. However, this treatment regimen may be promising for patients with *C. trachomatis*-positive and *U. urealyticum*-positive NGU. If the AZM ER 2 g single dose regimen fails to cure patients with *M. genitalium*-positive NGU, sitafloxacin (STFX), one of the newer oral fluoroquinolones has strong clinical activity against *M. genitalium* [29,30]. Therefore, the STFX 100 mg twice daily seven days regimen can be an effective alternative to the AZM ER 2 g regimen for patients with *M. genitalium*-positive NGU.

Although diarrhea was the main side effect, with a manifestation rate of 35.2%, it occurred temporarily and no patients with diarrhea needed additional treatment. A previous report clarified that gastrointestinal side effects, mainly nausea and vomiting, occurred in 35.3% of patients who received AZM IR 2 g, with a moderate degree in 10.1% and a severe one in 2.9% [11]. Although the frequency of side effects in the patients who received AZM ER 2 g was not very low, they were acceptable enough because of their temporary nature and limited degree. 

This clinical study has several limitations. The relatively small sample size might not have adequate statistical power. Some patients with treatment failure were lost to follow-up and we could not obtain the results of second-line treatment. Unfortunately, the sample number for *M. genitalium* was relatively small and it was hard to determine the exact treatment efficacy of the AZM ER 2 g single dose against this pathogen. However, this is the first clinical study to evaluate the clinical efficacy of AZM ER 2 g oral single dose treatment for patients with GU and NGU. This study demonstrated the excellent treatment efficacy and adequate tolerability of the oral 2 g single dose AZM ER regimen. A future randomized controlled study with a larger series will be able to further clarify the treatment efficacy for patients with GU and NGU. 

## 5. Conclusions

The treatment regimen with a single 2 g oral dose of AZM ER is effective for patients with GU and NGU. Although the frequency of side effects was 35.2%, these side effects were acceptable because they were mild and temporary, and this treatment regimen could be used safely. However, the antimicrobial susceptibilities of *N. gonorrhoeae* and *M. genitalium* and clinical efficacy in patients with GU and NGU should be carefully monitored because of possible treatment failure of AZM in the future.
